# Analysis of Pipeline Steel Corrosion Data From NBS (NIST) Studies Conducted Between 1922–1940 and Relevance to Pipeline Management

**DOI:** 10.6028/jres.115.026

**Published:** 2010-10-01

**Authors:** Richard E. Ricker

**Affiliations:** Materials Science and Engineering Laboratory, National Institute of Standards and Technology, Gaithersburg, MD 20899

**Keywords:** corrosion, pipeline, pipeline corrosion allowance, pitting, statistics, steel

## Abstract

Between 1911 and 1984, the National Bureau of Standards (NBS) conducted a large number of corrosion studies that included the measurement of corrosion damage to samples exposed to real-world environments. One of these studies was an investigation conducted between 1922 and 1940 into the corrosion of bare steel and wrought iron pipes buried underground at 47 different sites representing different soil types across the Unites States. At the start of this study, very little was known about the corrosion of ferrous alloys underground. The objectives of this study were to determine (i) if coatings would be required to prevent corrosion, and (ii) if soil properties could be used to predict corrosion and determine when coatings would be required. While this study determined very quickly that coatings would be required for some soils, it found that the results were so divergent that even generalities based on this data must be drawn with care. The investigators concluded that so many diverse factors influence corrosion rates underground that planning of proper tests and interpretation of the results were matters of considerable difficulty and that quantitative interpretations or extrapolations could be done “only in approximate fashion” and attempted only in the “restricted area” of the tests until more complete information is available.

Following the passage of the Pipeline Safety Improvement Act in 2002 and at the urging of the pipeline industry, the Office of Pipeline Safety of the U.S. Department of Transportation approached the National Institute of Standards and Technology (NBS became NIST in 1988) and requested that the data from this study be reexamined to determine if the information handling and analysis capabilities of modern computers and software could enable the extraction of more meaningful information from these data. This report is a summary of the resulting investigations.

The data from the original NBS studies were analyzed using a variety of commercially available software packages for statistical analysis. The emphasis was on identifying trends in the data that could be later exploited in the development of an empirical model for predicting the range of expected corrosion behavior for any given set of soil chemistry and conditions. A large number of issues were identified with this corrosion dataset, but given the limited knowledge of corrosion and statistical analysis at the time the study was conducted, these shortcomings are not surprising and many of these were recognized by the investigators before the study was concluded. However, it is important to keep in mind that complete soil data is provided for less than half of the sites in this study. In agreement with the initial study, it was concluded that any differences in the corrosion behavior of the alloys could not be resolved due to the scatter in the results from the environmental factors and no significant difference could be determined between alloys. Linear regression and curve fitting of the corrosion damage measurements against the measured soil composition and properties found some weak trends. These trends improved with multiple regression, and empirical equations representing the performance of the samples in the tests were developed with uncertainty estimates. The uncertainties in these empirical models for the corrosion data were large, and extrapolation beyond the parameter space or exposure times of these experiments will create additional uncertainties.

It is concluded that equations for the estimation of corrosion damage distributions and rates can be developed from these data, but these models will always have relatively large uncertainties that will limit their utility. These uncertainties result from the scatter in the measurements due to annual, seasonal, and sample position dependent variations at the burial sites. The data indicate that more complete datasets with soil property measurements reflecting the properties of the soil and ground water directly in contact with the sample from statistically designed experiments would greatly reduce this scatter and enable more representative predictions.

## 1. Introduction

Currently, the U.S. has over 3.7 million kilometers (2.3 million miles) of pipelines crossing the country transporting natural gas and hazardous liquids from sources such as wells, refineries, and ports to customers. It is estimated that almost 2/3 of the energy consumed in the U.S. passes through a pipeline at some point between its origin and the point of consumption and that pipelines account for about 20 % of the total mass-distance that oil and natural gas are transported [1, 2]. Clearly, the maintenance of an uninterrupted energy supply to the public requires the operation of these pipelines in such a manner that corrosion does not result in an unscheduled interruption to the flow of these energetic materials to the nation, as occurred recently in Alaska [3]. This task is accomplished by pipeline operating companies, who follow standards, codes, and practices set out by a variety of regulatory agencies, industrial consortia, and standards developing organizations. The Pipeline Standards Developing Organizations Coordination Council (PSDOCC) coordinates the activities of these groups, and the Department of Transportation’s Office of Pipeline Safety (OPS) is the main regulatory agency with final responsibility over this system of codes and practices [2].

Following pipeline accidents in Carlsbad, NM [4] and Bellingham, WA [5], the U.S. Congress passed the Pipeline Safety Improvement Act of 2002 (PSIA). The objective of this act was to improve public safety by stimulating improvements in pipeline technologies, regulations, and standards. This act resulted in the formation of the PSIA Coordination Council, which communicates and coordinates pipeline relevant research in four government agencies: The Department of Energy, The Department of Transportation, The Department of Interior, and The Department of Commerce. This project is a result of this collaboration. The objectives of this project were to (1) reexamine the original NBS underground bare pipe corrosion studies to determine if the results from this study could be used to develop better empirical models for prediction of bare pipe corrosion rates and (2) to seek new in-sights that could lead to the development of pipeline external corrosion prediction models, or soil corrosivity indexes, that could be used in the future for computer-aided pipeline management.

**Fig. 1:**
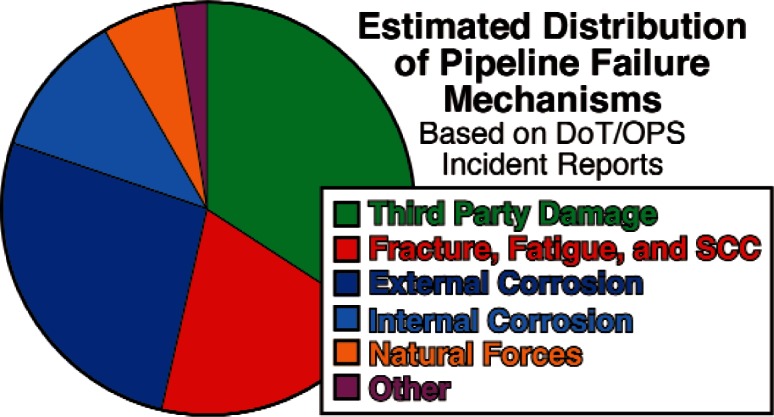
Attributed failure mechanisms for reported pipeline failures.

**Fig. 2:**
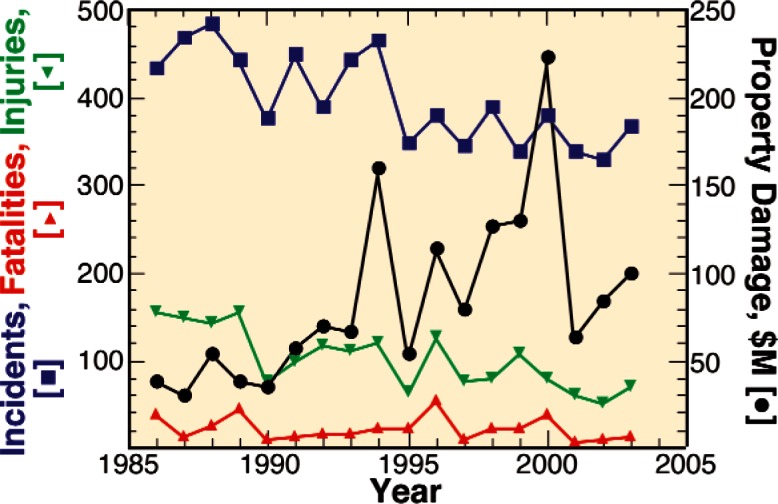
Statistics from the Office of Pipeline Safety on pipeline accidents.

Since the mileage of existing pipelines greatly exceeds that of new construction, the average age of the U.S. pipeline infrastructure is increasing steadily [6]. Penetration of the pipeline wall as a result of corrosion of the external surface is responsible for a significant portion of pipeline failures as shown in Fig. 1 [6]. Assuming that corrosion rates are greater than zero, this means that the threat of corrosion-induced failures is actually increasing steadily each year. Considering this, it is surprising that this industry has been able to actually reduce or hold failure rates constant over recent years as shown in Fig. 2 [6]. This feat has been accomplished through the accumulation of pipeline operation experience, and improvements in technologies including inspection, repair, coating, and information technologies. This industry openly shares their experience through a number of different consortia and standards developing organizations. As a result, the practices, codes, and standards developed reflect this experience and evolve as pipelines age and new technologies are developed. This has enabled this industry to make improvements and repairs before failures actually occur. This industry can be expected to make further improvements as better inspection, repair, and information technologies are developed that allow this industry to monitor, measure, and track changes in their pipelines to even greater resolutions and detail.

It is conceivable that in the future pipeline operators will have computer systems that provide data on every meter of the pipelines in their system at their fingertips. Ideally, one of the parameters for each increment of the pipe will be an indicator of the corrosivity of the local environment to the steel of the pipeline wall. This measure may be based on sensor readings or estimated from measures of soil properties and chemistry. This parameter will give the operator information on how long the steel pipe should be able to contain its contents without catastrophic failure should the coating and/or cathodic protection systems fail. Ideally, this parameter will help the operator schedule inspections and plan shutdowns for repairs so that the energy supply is uninterrupted. The quality of the decisions that operators will make based on this parameter will depend on the accuracy or the uncertainty in the estimate of this parameter. Currently, operators are required to use the same “corrosion allowance” for all corrosion rate based decision-making unless they can prove that a lower corrosion rate can be expected.

The value used for the current corrosion allowance was determined by analysis of underground corrosion measurements taken by the National Bureau of Standards (NBS) during a study conducted between 1920 and 1947 [7, 8]. The same value is to be used for all soils and underground pipeline environments without regard for the specific soil chemistry of each site and local conditions. There is a provision for exceptions when an operator can demonstrate that lower rates can be expected for a particular section. This is a conservative approach at present that grows more conservative as information and other technologies improve. Advances in computers, sensors, chemical property measurements, and computer modeling of chemical reactions and transport can be expected to make this an overly conservative approach in the near future. These emerging technologies will make the acquisition and manipulation of increasingly detailed information on increasingly smaller increments of a pipeline possible. The first step toward accomplishing this next level of corrosion allowance determination should be the establishment of a link between some measurable property of the pipeline soil environment and the resulting corrosion rate. There are essentially three different approaches that can be taken to establish this link: (1) empirical correlations to actual measurements of corrosion damage in steel pipes exposed to representative soils, (2) development of laboratory measurements and models for estimation, and (3) detailed computer models with valid assumptions for rate determining processes. Each of these different approaches has advantages and drawbacks, but all three will require verification with actual data from exposure tests on samples in representative soil environments. Therefore, the first of these is the logical starting point especially since it will help one identify the critical issues for the other two.

Information and data on the corrosion behavior of steels in underground environment is rather limited, and many studies into underground corrosion rely on the data from the studies conducted by NBS between 1920 and 1947 [7, 8]. The data from these studies have been used for underground corrosion decision making over a wide range of fields from underground utilities to nuclear waste disposal. These studies actually began in 1911 when Congress asked NBS to conduct studies into electrolysis failures caused by the operation of electric streetcars. In conducting this study it was noted that very little was known about how steels and other metals should corrode underground in the absence of induced electric currents induced from the operation of streetcars. As this study was nearing completion, it was noted that the emerging pipeline industry had a critical need for this type of information. As a result, a workshop was held at NBS with participants from industry and an underground corrosion research program was proposed. The Department of Agriculture was asked to identify locations with representative soils and to participate in the characterization of the soils at the sites. Industry was asked to provide samples and to participate in sample burials, removals, and inspections. Workshops were convened at regular intervals to keep everyone updated and were attended by corrosion experts from all over the world. This study lead to a large number of similar studies of corrosion in real world conditions and eventually into the development of related laboratory research programs in corrosion measurement methods at NBS that evolved over time into the present programs in the Metallurgy Division of NIST.

**Fig. 3:**
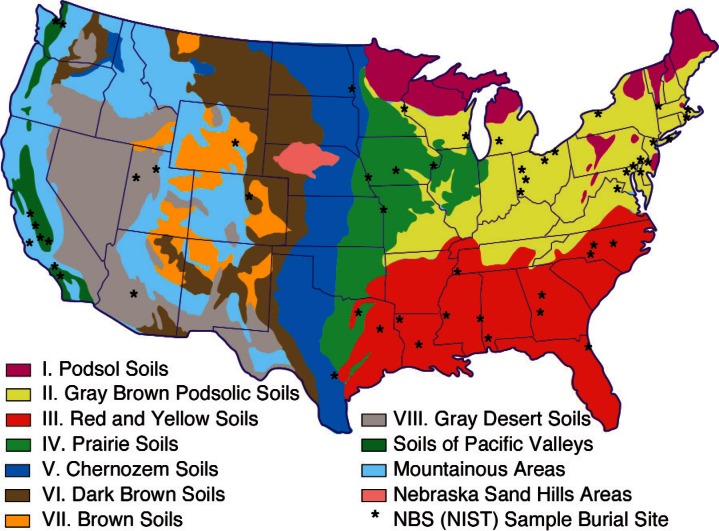
Map of the US showing the locations of the burial sites and the 8 major soil groups identified in the study.

**Fig. 4:**
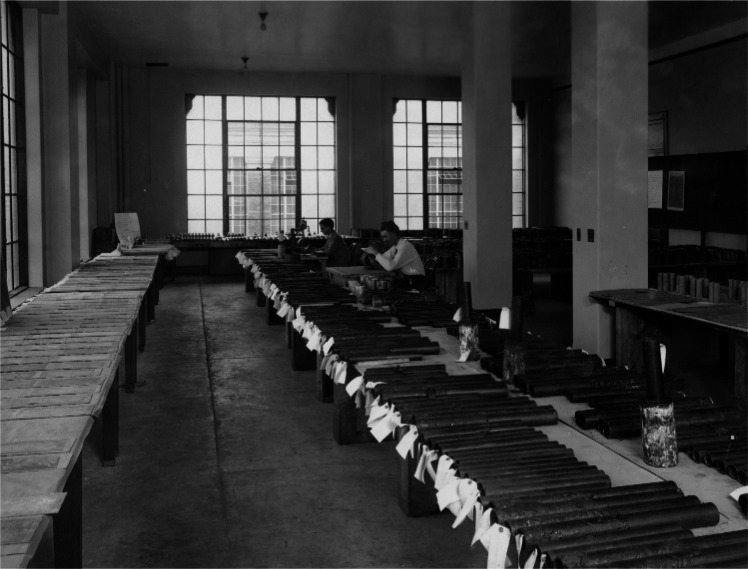
Samples being examined in the laboratories at NBS.

The original NBS bare pipe underground corrosion studies incorporated 47 sites across the United States as shown in Fig. 3. In this figure, the 8 basic soil types are identified as they were in the 1957 summary report. Since the 1957 report was prepared, the Department of Agriculture has subdivided soil groups and currently lists 13 major soil groups in the United States [9]. Detailed soil maps with these updated classifications can be obtained from the Department of Agriculture [9]. Samples were retrieved from sites at periodic intervals, with the last samples removed between 12 and 17 years after burial depending on the site. Figure 4 is a photograph of the samples from this study laid out in the NBS laboratory for examination. The bare pipe corrosion study was the first of a long series of studies of corrosion in real world situations conducted by NBS [7, 8].

When NBS began the underground corrosion studies of bare steel pipes (1922–24), the Department of Agriculture identified sites for the placement of coupons and conducted soil surveys to characterize the soils. Soil samples were then analyzed by NBS to determine the composition and properties of the soils at the sites. Soil surveys and taxonomy were new concepts just being developed in the 1920s [9]. The soil property measurements and chemical analyses were also state-of-the-art for the time the study was conducted. Statistical analysis was not a well developed and appreciated part of metrology when these studies were designed. The NBS underground corrosion studies have been criticized for the poor statistical design of the experiments including neglecting the distribution of the samples at the sites [10]. That is, soil horizons, while mostly parallel to the surface, frequently vary in depth even over the short distance of a burial trench. As a result, samples from opposite ends of the same trench could be exposed to different conditions. A well designed experiment for statistical analysis would have the samples distributed in the trenches in a manner that avoids this spatial bias. In addition, seasonal and annual bias can result from variations in starting dates, exposure times (fractional years), and the use of average annual data for conditions rather than measurements.

The original NBS underground pipeline corrosion study appears to have attempted to mimic the pipeline burial conditions and practices of the day for each location (e.g., burial depth varies with location). In this manner, the results would represent the uncertainties inherent in these practices and conditions rather than just fundamental information on the influence of soil chemistry and properties on corrosion rates. For example, the annual rainfall given for each site is actually the average annual rainfall for the location closest to the burial site with rainfall data and not an average for the actual site or the years of burial. At the time of the study, this was the only kind of information that would be available to a pipeline operator and remote sensing, recording, or monitoring was not to be a consideration for decades. In addition, many soil properties were measured in the laboratory rather than in the field. Removing soils from the ground will alter the activity of important species such as water, carbon dioxide and oxygen and alter the activity of biological species and the properties of the soils. The impact of these factors on pH was recognized by the 1950s and Romanoff attempted to correct the pH values [8]. In addition, cost appears to have been a factor during the studies limiting site selection, sample layout, and examination. Of particular concern is the fact that chemical analyses were conducted on soils from only 26 of the 47 sites. Today, statistical analysis considerations would dictate that all sites should be characterized or that the 26 sites should be selected at random. However, the sites with soil chemistry data are the 26 with the lowest measured electrical resistivity; and therefore, the highest concentrations of soluble salts. These issues should not be considered the fault of the original investigators because their importance in obtaining data for statistical analysis was not fully appreciated at the time these studies were designed. It appears that the original study decided to emulate the buried pipeline conditions, soil characterization data, and the associated uncertainties inherent in the information that would be available to pipeline operators. This would mean that the resulting data would have greater scatter than might result for more controlled conditions, but this scatter would represent the “real-world” uncertainties that pipeline regulators and operators would confront when making decisions. Including this scatter in the data insured the data would be representative and that decisions made would be conservative for the prevailing conditions of the day. Decades later, this seems to be an overly conservative approach that inhibits statistical analysis, interpretation, and the development of performance prediction models.

In the near future, information technologies, sensors, and global information systems (GIS) will make it possible to characterize or even monitor environmental chemistries and soil properties at closely spaced intervals along a pipeline. Not only will pipeline operators have larger quantities of better soil characterization measurements, they will have better tools for manipulating and interpreting this information. Computer aided monitoring, data manipulation, and operation decision-making is becoming standard practice. All of these possibilities were not even a consideration in the 1920s when the original NBS study was initiated. In fact, when the study initiated it was not even clear that coatings would be required to protect pipes from corrosion. Today, cathodic protection and coatings are used extensively. One of the most significant impacts of the original study may have been to determine that coating would be required and to stimulate coatings research and development. Coatings for pipeline protection and pipeline coating technologies are still a major area of research and development today [11].

For the analyses of this study, it will be assumed that the soils removed from the trench were used for backfill and that the chemical and physical properties given in the summary reports accurately represent the soil in physical contact with the samples at each site [7, 8]. Since there is no information on uncertainty or variability in the reports [7, 8], thorough homogenization to these precise values must be assumed. Similarly, without seasonal data, it must be assumed that there are no seasonal variations in conditions and corrosion rates that would allow for the exact dates of placement and retrieval and fractional years of exposure to have an influence on the results. Also, it must be assumed that the years of burial were typical years so that the annual rainfall, temperature, and other soil characteristics are properly represented by the data. The additional uncertainty imposed by these assumptions should be kept in mind along with the conclusion in the 1957 summary report that the statistical variability in the data was too great to make reliable predictions possible [8].

## 2. Burial Site Characterization

The term soil is usually used to describe any of the naturally occurring loose collections of solid particles found on the surface of the earth that support the growth of plants [9]. This includes the inorganic minerals, organic species, liquids, and gasses found in these aggregates. According to a strict interpretation of this definition, a soil only extends as deep as the roots of the plants or other organic species that grow in the soil. Today it is understood that soils are alive with organic species of all types and sizes [12]. Pipelines are typically buried below the levels where these organic species are plentiful and special backfill free of organics may be used rather than the dirt and soils removed from the trench. This does not mean that microorganisms will not influence the corrosion rate of a pipeline. Sulfatereducing bacteria have long been known to stimulate corrosion of steel pipelines in anaerobic environments with sufficient nutrient content [13, 14]. In addition, biological activity both in the soils above the pipeline as well as those above the surface of the soil may have a dramatic influence on the water, oxygen, and carbon dioxide content at the burial depth of the pipe. However, these factors were not quantified for examination in the original NBS study and cannot be considered here. In addition, it will be assumed that the backfill was the soil removed from the trench and that the chemical analysis of the soil at each site provided in the summary reports covering these studies presents a reasonable estimate of the chemical environment the samples experienced during these exposures [7, 8].

**Fig. 5:**
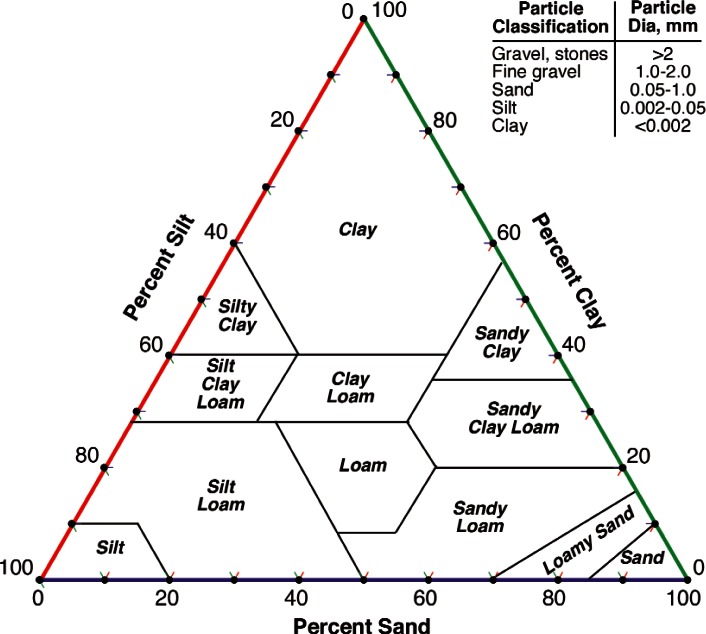
Ternary diagram describing soil types by characteristic particle sizes.

Soils are composed of essentially four features (1) mineral particulates, (2) organic matter from surface and subsurface plant and animal life, (3) groundwater containing soluble salts, and (4) gases. The particulate matter found in soils is usually small particles of the minerals found in the nearby rock formations that were produced from these formations over millions of years of weathering and the decomposition products produced when these minerals react with air and water. In either case, most of the particles making up a soil are insoluble minerals, as most soluble species have been removed over the millions of years of weathering. The solubility of these minerals may vary with pH, and if they do so, they will tend to buffer the pH of the groundwater, but assuming no significant changes in pH with time, we can assume for a first order approximation that these minerals behave as inert solids. Soils are placed into categories as sands, clays, silts, or loams based on the size distribution of these particles as shown in Fig. 5. The potential influence of organic matter either living and excreting potentially corrosive compounds or decaying and producing potentially corrosive conditions locally should not be ignored in a thorough life prediction scheme, but information on these conditions were not collected with the data of these studies. It is also important to realize that the properties assigned to a site may change over time due to human, animal, or plant activity, but there is also no information on these types of changes occurring at the burial sites.

It should be kept in mind that the objectives of the original NBS study were (1) to determine if bare pipe could be used in some or all soils and (2) to determine if measures of soil characteristics could be used to predict the corrosivity of soils and enable better pipeline decision-making and management. To accomplish the second objective, one might want to include all of the natural range of variability that could be expected for normal pipeline burial practices of the day, since it is the extreme rates that will produce failures. At the time these studies were conducted, data were manipulated and analyzed manually and a single soil sample might be used to represent the soils and exposure conditions for a considerable length of pipeline. Since at that time statistical tools for addressing these issues were very limited, it is logical that one would want to include the complete natural range of actual conditions that a single set of soil property data might be used to represent. Any attempt to control or limit this natural variability might be viewed as producing data less representative of “real-world” industrial practice since it would not include the entire range of conditions and rates expected for a soil with the properties indicated by the soil sample.

The sites for burial of the samples were identified by the Department of Agriculture and they were selected to represent the different types of soils and conditions that could be found in the U.S. The sites were identified by number, location, and soil type as shown in Table A1 (Appendix A contains the tables of site descriptions and measured characteristics). Table A2 lists the 26 different parameters used to identify or represent the properties of the soils found at the burial sites along with the units used in the original reports and the current SI equivalent units with the conversion factors used for this study. Some measures were arbitrary ratings such as fair, good, and poor for site internal drainage, some were measures of soil properties, and some were taken from locally available data such as average annual rainfall and ground temperature. Most of the soil properties were measured for all 47 sites (Tables A3 and A5), but the chemistry of water extract was determined for only the 26 sites with the lowest resistivity measurements (Tables A4 and A6). Table A7 contains the complete descriptions of the soil horizons and depths for all of the sites used for this study.

The relationships between electrical conductivity of the medium or electrolyte and corrosion behavior have been the subject of much debate and some research [15–18]. The conductivity of an electrolyte is the product of the concentration of charge carrying species and their mobility. The ionic bonding of the insoluble mineral particles will prevent conduction through these particles. Therefore, electrical conduction will be restricted to the solutions in the pore spaces around the particles and the conductivity of the water-saturated soil is a measure of the soluble salts present in the soil to form ions in the water, the volume fraction of pore space, and the mobility of the charge carrying ions. Fortunately, most ions other than the hydrogen and hydroxide ion have similar mobilities in aqueous solutions. So, conductivity is simply an estimate of the total ion content of the solution surrounding the particles of soil. In general, corrosion rates are observed to increase with the conductivity of a soil. Increasing the conductivity of the electrolyte enables greater separation of the cathodic and anodic half-cell reactions. It also reduces the range of potential differences that are possible between different sites on the surface of the sample. Escalante et al. [15–18] examined the effects of conductivity, temperature, and mass transport in soils and found that while lower conductivities tended to result in lower average corrosion (mass loss) rates, they also resulted in a greater range of variations in corrosion rates across the surface. This would result in increased pitting ratios that could result in wall penetration rates equivalent to those of more corrosive environments with lower pitting ratios. That is, while lower conductivities (higher resistivities) tend to result in lower overall corrosion rates, it also makes it easier for corrosion to localize to a small spot or region of the surface and form pits. This was observed and reported in the original NBS studies and identified in the summary reports as a major factor contributing to the scatter in the data that made reliable corrosion predictions difficult [7, 8].

**Fig. 6:**
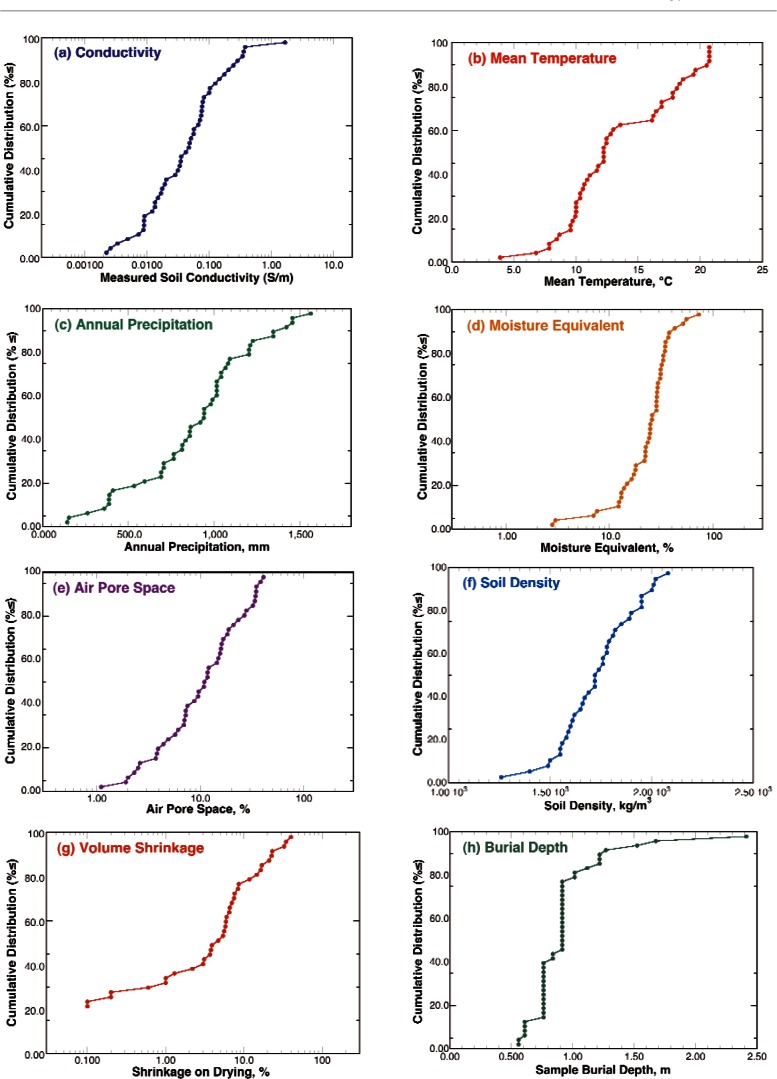
Cumulative distributions functions for the measured properties of the soils.

**Fig. 7:**
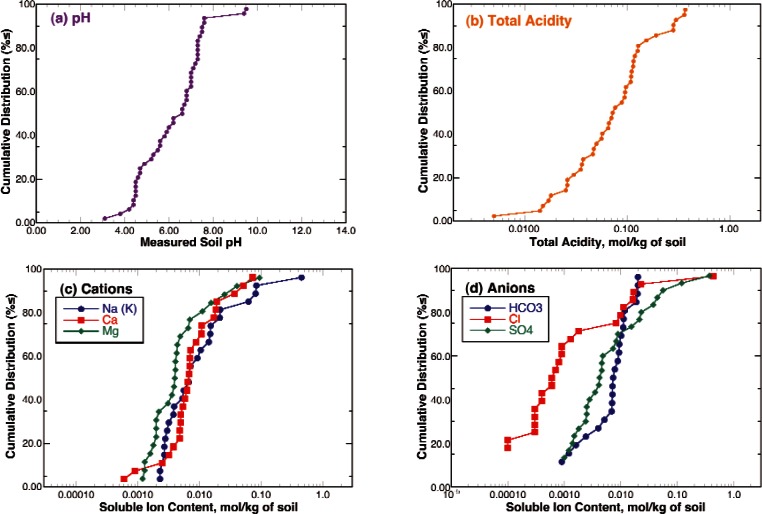
Cumulative distribution functions for the concentrations of soluble chemical species in soils and total acidity: (a) pH, (b) total acidity, (c) cations, and (d) anions.

The first step in analyzing the data from the NBS bare pipe underground corrosion study was to plot cumulative distribution functions (CDF) for the measurements characterizing the properties of the soils at the sites (Table A3) as shown in Fig. 6. In these figures, the x-axis is the measured property and the y-axis is the fraction or percentage of sites having this value for the property or less. In this manner the CDF goes from 0 to 1 or 0 % to 100 % over the measured range for the variable. The slope of the CDF is the more familiar probability density function (PDF) or the fraction of sites within some bandwidth of the value given on the x-axis (i.e., density). A log-normal distribution was used for all measures of chemical concentrations or measures that would relate to chemical reactivity, as chemical reaction kinetics typically vary with the log of the activity or concentration of the reaction species (Table A4 and Fig. 7)[19]. The exception to this is pH, as it is a log scale.

A standard score (Z) was calculated for each characteristic (i) at each site (j) according to the relationship

(1) $$Z_{ij}=\left(\frac{x_{ij}-\mu_{x}}{\sigma_{x}}\right)$$

where *x_ij_* is the measured value of the characteristic for the normal distributions and the logarithm of the measurement for the log-normal distributions and *μ_x_* and *σ_x_* are the corresponding mean and standard deviation values of this property. Converting the measurements to a standardized variable (Tables A5 and A6) produces scores for analysis that are without units and can be compared on the same graph with the same scale without bias. Conversion from the Z-score back to original units is simple matter of applying the mean and standard deviation given in Tables A5 and A6 through Eq. (1) above.

In addition to the measured physical and chemical properties of the sites, the depth and nature of the soil horizons (horizontal layers or strata) were qualitatively characterized, and these are included along with the depth that the samples were buried in Table A7. This table is included to illustrate the complex nature of the soils at the sites and to demonstrate how the behavior of samples from one part of a site to another could vary if depth of the horizons varied.

## 3. Corrosion Damage Characterization

Appendix B contains the tables of corrosion damage measurements. In addition, Table A2 includes measured units and conversions used for the measures of corrosion damage along with those for the site characterization parameters. Corrosion damage was characterized by measuring two factors: (1) mass change and (2) pipe wall thinning. The mass change was measured after the corrosion products were removed in a manner such that the underlying metal would remain intact. The descaling procedures used in the studies are described in the 1945 report [7]. In addition to the average mass change for two samples, the average of the deepest penetration into the wall of two pipes was reported. This results in two measures of damage for the exposure: (1) mass loss and (2) corrosion penetration. These two can be converted to rates by dividing by the exposure time. This calculation assumes that the corrosion rates are effectively constant over the exposure time. Of course, mass loss can be converted to an average penetration rate using the density of the metal as shown in Table A2. The ratio of the maximum measured penetration to the average penetration calculated from mass loss is the pitting ratio, which is a measure of the propensity of the exposure environment to cause local variations in the corrosion rate over the surface of a sample (a pit being a high corrosion rate at a small spot). In this study, essentially three corrosion response variables were studied: (1) the mass loss rate (MLR), (2) the corrosion penetration rate (CPR), and (3) the pitting ratio. As with the environmental variables, the units and conversion for these measures are given in Table A2.

Eight different types of samples were buried at each sight with 6 sets of duplicates for periodic retrieval. The samples were provided as nominal 1.5 in and 3 in pipe (38.1 mm and 114.3 mm). Table B1 identifies sample size and alloy composition by the single letter used to identify each sample type: “a,” “b,” “e,” “y,” “B,” “K,” “M,” and “Y”. The alloys and microstructures of these samples almost certainly deviate significantly from those available today primarily due to the dramatic improvements in processing that has reduced slag inclusions and mill scale. Apparently, in an effort to accurately represent the conditions of actual buried pipeline, no special effort was put into cleaning sample surface and removing mill scale beyond that required to remove oils and allow sealing of the ends with caps. Mill scale and inclusions are typically noble with respect to the Fe of the metal and the presence of these phases on the surfaces will stimulate cathodic activity enabling higher corrosion rates that might be localized to the region around these phases depending on the nature (conductivity) of the surrounding soil. In addition, the graphite phases in the microstructure will also tend to act as sites for cathodic (reduction) reactions, and the finer more controlled microstructures available today will reduce the tendency of these features to localize corrosion. However, without hard data on these differences and their impact, it must be assumed that these alloys represent the range of alloys used in pipelines past and present.

The logistics and cost of maintaining exact year exposure increments in these studies outweighed the desire for data from identical exposures. If there is a seasonal variation in the corrosion rate, it will contribute to the unquantifiable scatter in these measurements that cannot be explained since there are insufficient data on the dates of burial and retrieval. This is significant because it is possible that a site may have rainy and dry seasons such that almost all of the “annual corrosion damage” occurs in one season. In this case, an exposure of 1.25 y could have twice the damage of an exposure of 1.0 y. Seasonal variations are frequently observed in real world exposure tests, but these cannot be addressed with the current dataset.

As pointed out in the section above on site characterization, the corrosion damage to the samples was quantified by measuring the change in the mass of the samples over the burial period and by measuring the maximum depth of wall penetration in the samples. The mass loss was measured after removal of corrosion products and the descaling techniques are described in the 1945 summary report. The mass loss was reported as the average mass loss per unit area (oz/ft^2^) for two samples of each of 8 different types of ferrous pipeline alloys. These measurements converted to the current SI units for mass loss (g/m^2^) are presented in Table B2. The exposure times were given with the mass loss data and converting these to mass loss rates in grams per meter squared per day (g/m^2^/d) results in the data presented in Table B3. Similarly, the maximum corrosion penetration measurements were presented as the average maximum depth of penetration (mils) for two samples. These measurements converted to SI units (mm) are given in Table B4 and after conversion to penetration rates (mm/y) in Table B5.

**Fig. 8:**
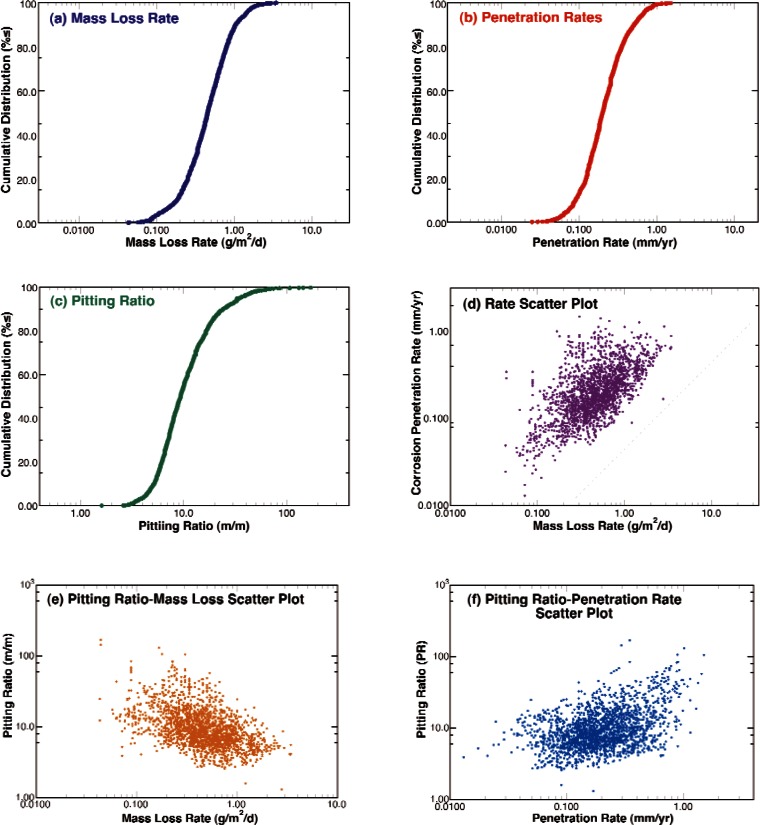
Cumulative distribution functions for the corrosion mass loss (a) and penetration rates (b) and the normalized ratio of these rates or pitting ratio (c). Scatter plots examining the relationships between these measures of corrosion damage rates: (d) penetration v. mass loss, (e) pitting ratio v. mass loss, and (f) pitting ratio v. penetration.

**Fig. 9:**
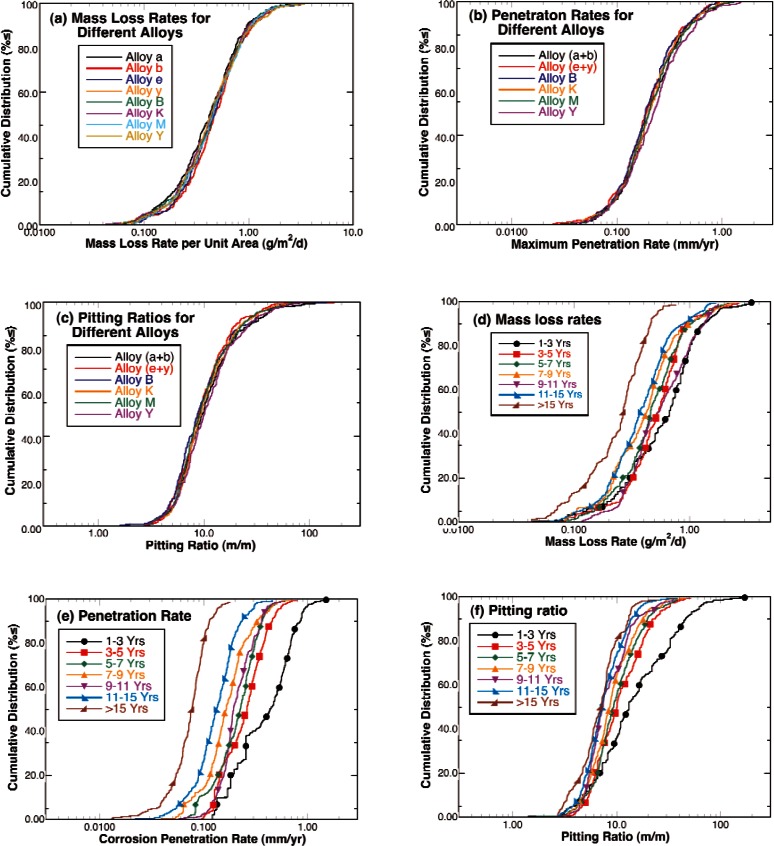
Cumulative distribution functions examining the effects of alloy composition and exposure time on the measurement: (a) Mass loss rates for different alloys, (b) Corrosion penetration rates for different alloys, (c) Pitting ratios for different alloys, (d) Mass loss rates for different retrieval periods, (e) Corrosion penetration rates for different retrieval periods, and (f) Pitting ratios for different retrieval periods.

The cumulative distribution functions for the corrosion mass loss rates, penetration rates, and pitting ratios are shown in Figs. 8(a) through 8(c). These figures show that combining the measurements from all of the sites results in a smooth and symmetric sigmoidal curve when plotted as the log of the rate or ratio indicating that log-normal distributions can be used to represent these data. Plotting these measures against each other with log scales as in Figs. 8(d) through 8(f), shows that there is no clear trend relating these measures and that the data form ellipsoidal scatter plots. Segregating the data into subgroups based on alloy type results in the cumulative distribution functions shown in Figs. 9(a) through Figs. 9(c). By examining these figures, it is clear that scatter in the measurements resulting from the exposure variables and the natural stochastic nature of underground corrosion overwhelms any differences due to alloy type for this range of alloy compositions. This is not uncommon for steels [20, 21]. Therefore, subsequent analyses will assume that measurements from these alloys can be considered to be from the same alloy and analyzed as such to add numbers and statistical weight to the trends. On the other hand, breaking the measurements into subsets according to the length of time that the samples were underground indicates that both mass loss rates and corrosion penetration rates decreased with the time that the samples were buried in the ground. In addition, the pitting ratios also tended to decrease with exposure time. This is an important observation that will be discussed later in this report.

Since the exposed surface area of the samples could influence the observed maxima in penetration depth and sample types “a,” “b,” “e,” and “y” had almost exactly half the exposed area of sample types “B,” “K,” “M,” and “Y,” subsequent analyses of the corrosion penetration rates were done by taking the maximum reported for sample types “a” or “b” as a single measurement and similarly the maximum for sample types “e” and “y” as a single measurement. As a result, there are 6 measurements of maximum corrosion penetration rate per site and retrieval while there are 8 measurement of mass loss rate.

**Fig. 10:**
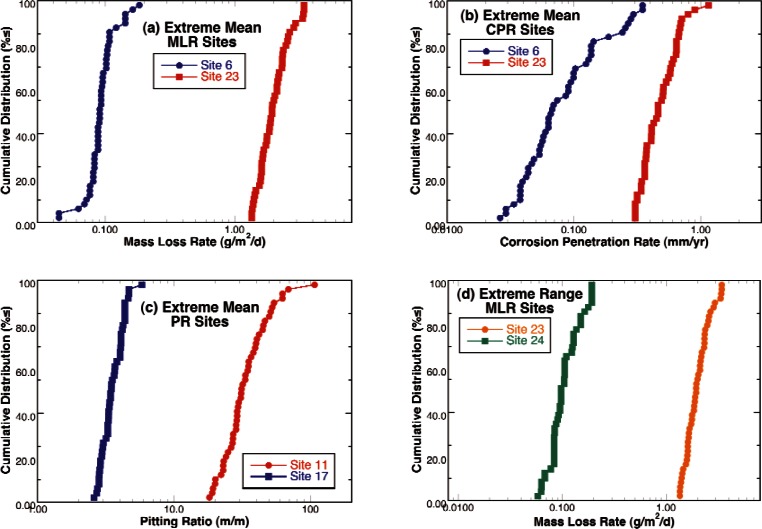

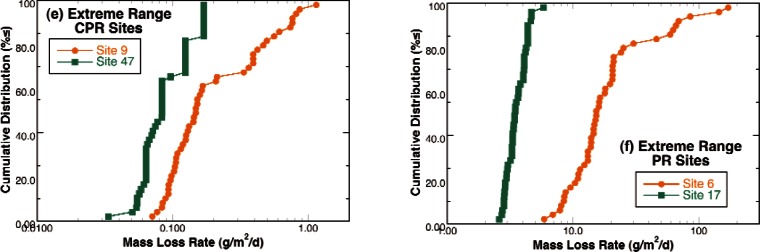
Cumulative distribution functions for sites illustrating the range of behavior observed. Sites exhibiting minimum and maximum (a) mean mass loss rate, (b) mean corrosion penetration rate, and (c) mean pitting ratio and sites with the minimum and maximum range for (d) mass loss rates, (e) corrosion penetration rates, and (f) pitting ratios.

To briefly illustrate the range of variations among the different exposure sites for these measures of corrosion damage, sites representing the extreme maximum and minimum for the mean and range of these 3 corrosion measures are plotted in Fig. 10. Figure 10(a) illustrates the motivation for this and similar studies of corrosion damage rates. This figure shows that the maximum mass loss rate observed on any sample at Site 6 is at least an order of magnitude lower than lowest rate observed for any sample at Site 23. Clearly, these corrosion rates depend strongly on the characteristics of these sites and identifying the characteristics that can be used to reliable identify which range of behavior a pipeline will exhibit will enable better management decision making. However, Fig. 10(b) illustrates one of the main problems for accomplishing this objective. This figure shows the maximum penetrations observed for the samples at the same two sites shown in Fig. 10(a). The corrosion penetration rate distributions for these two sites overlap. Since these two sites represent the extremes in the mean log penetration rate, all of the other sites fall between these two and also overlap. This clearly illustrate the trend for the sites with lower mass loss rates to have a greater range of corrosion rates over the surface area of the samples resulting in more localized high rates or pitting. The sites representing those with the greatest and smallest range in the three measures of corrosion damage are shown in Figs. 10(d) through 10(f). Again, the mass loss rate measurements indicate over an order of magnitude difference with the variation being in proportion with the differences in the means creating two nearly parallel lines on the log scale. On the other hand, the corrosion penetration rates and pitting ratios shown in Figs. 10(d) and 10(e) do not form smooth continuous curves, but show irregular “jumps” in the curves indicating that samples above and below these “discontinuities” experience different conditions or that stochastic variations in processes resulted in the nucleation or creation of highly corrosive conditions. The discontinuity, rather than a gradual slope change, suggests that there is a threshold or nucleation event that separates the behavior of the pit from that of the remainder of the surface.

## 4. Environment-Corrosion Rate Relationships

**Fig. 11:**
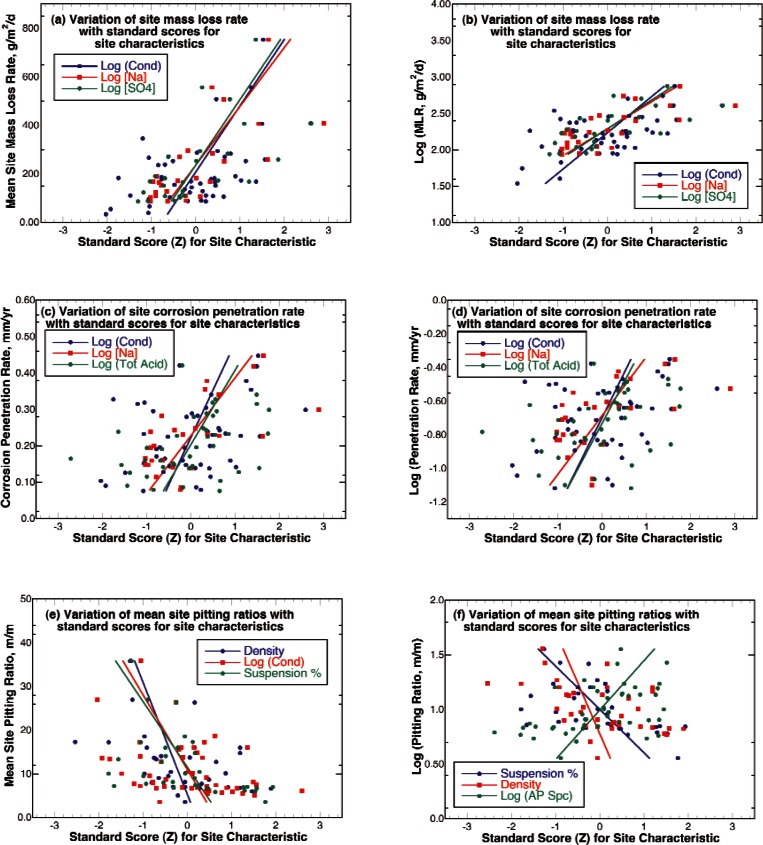
Linear regression results for fitting (a) site mean mass loss rates (MLR), (b) Log (MLR), (c) site mean corrosion penetration rates (CPR), (d) Log (CPR), (e) site mean pitting ratios (PR) and (f) log (PR) for selected site characterization parameters (see Tables C1 and C2).

The relationship between the three measures of corrosion damage and the quantitative variables describing the properties and chemistry of the soils at the sites were explored by plotting the standard score for the variables at the site against the corrosion damage measure and performing linear regression on the measurements using commercially available curve fitting software. Some of the better results from this regression process are illustrated in Fig. 11. By examining this figure, it can be seen that none of the variables exhibited well-defined trends with any of the corrosion measures. The correlation coefficient for a curve fit is the ratio of the unexplained variation to the explained variation; and therefore, is 0 when there is no indicated relationship between the parameters and has a magnitude of 1 when the curve fit can describe the exact location of every point. The correlation coefficients for the fit of these site characterization variables to the corrosion damage measures are given in Table C1. This process was repeated taking all of the samples for all of the sites as individual measurement points and the correlation coefficient for these fits are also shown in Table C1. By examining this table it can be seen that the best fit was found for the mass loss rate (log) as one might expect after examination of Figs. 8–10. The site characterization variables with the highest correlation coefficient were the concentrations of the ions Na^−1^ and SO_4_^−2^. The fits to the corrosion penetration rate were slightly lower with the same site characterizations variable resulting in the highest correlation coefficients. However, the highest correlation coefficient observed for any of the single variable regression fits was 0.714 that is not a particularly good fit as shown by the Na^−1^ line of fit in Fig. 11(a).

After examining linear regression fits, multiple regression analyses were performed on the site averages for the corrosion damage measures. Given the wide range of possible combinations and the number of variables, experimenting with scientifically logical and derivative fits proved to be a very time consuming and, given the poor quality of most fits, disappointing process. However, a scheme was developed and followed for the evolution of a fit. This scheme results in a completely empirical fit in that there is essentially no scientific consideration given to the selection of the variable used in the derivation of the fit other than it was selected for measurement in the original study. Basically, each corrosion damage measure was fit against each of the site characterization variables taking one at a time (single linear regression). Then the variable that produced the best fit was used in 2-term regression model using all of the remaining site characterization variables taking one at a time. This process was repeated for 3, 4, and 5 term multiple regression models. At each step, the site characterization variables yielding the second and third best correlation coefficient were examined in place of the best fit to insure that the best fitting variable was selected.

The exception to this process was soil conductivity. Since the conductivity of the soil is a measure of the total ion content of the soils, it is a measure of the combined concentrations of all soluble ions. Since ion chemistry was measured for only 26 of the 47 sites, using any of the ions concentrations in the fit significantly reduces the number of points being fit. This was considered undesirable particularly for the early terms in the process. In addition, when Na^−1^ fit well with a measure of corrosion, the anions Cl^−1^ and SO_4_^−2^ also showed higher correlations making it unclear which was the more important. Using conductivity, at last in the first term, allows for representation of ion concentrations without forcing an empirical selection of an ion that may not be important in determining the rate as much as simply varying with the ions that do matter. It should be kept in mind that these are totally empirical fits and may not even indicate the important variable as many of these site characterization variables are interrelated, and this empirical variable selection process may result in the selection of a variable that varies with an important property, but was measured or represents the causative property better for the sites than the measures used to quantify that property.

**Fig. 12:**
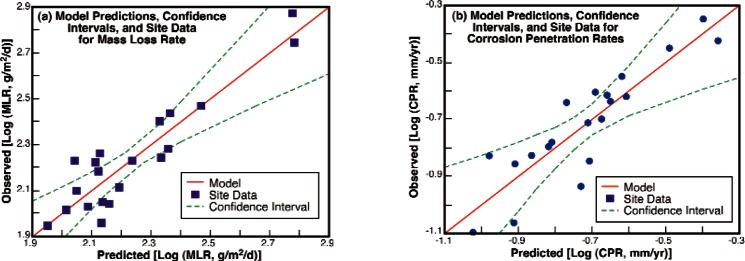
Multiple regression modeling results for mass loss rates (a) and corrosion penetration rates (b) as a function of site characteristics.

The result of this term-by-term multiple regression fitting process are given in Tables C3 and C4 for fitting the site average mass loss rate and corrosion penetration rates respectively. The predictive capability of these models is illustrated graphically in Fig. 12. The correlation coefficient for multiple regression fits for prediction of the site average mass loss rate was 0.942 and 0.956 for 5 and 6 terms, respectively. Similarly, the fits for the corrosion penetration rate yielded correlation coefficients of 0.860 and 0.891 for 5 and 6 terms. These empirical fits allow for estimation of the mean, average, or expected value for a site given the properties used in the calculation. The scatter in the fits allows for the estimation of the scatter that should be observed at a site characterized by the variables. That is, the uncertainty in the curve fit is illustrated graphically in Fig. 12 by the dashed lines for the confidence interval.

## 5. Variation of Rate with Exposure Time

As shown in Fig. 9, the mass loss rate and corrosion penetration rates tended to decrease with exposure time. A decrease in the corrosion rate with time is not unexpected as there are a number of different kinetic rate models that would predict such a trend [19–21]. First, if corrosion products build up on the surface and this inhibits the transport of reactants to or from the surface, the corrosion rate will decrease as this layer grows thicker if mass transport through this layer is rate limiting. Similarly, if there is a cathodic reactant that is being consumed by corrosion and it is being depleted from the surrounding environment, a slow decline in the corrosion rate with time is to be expected. In either case, the measured (average) corrosion damage rate will decrease with increasing exposure time. The behavior of the measured corrosion damage as a function of time will indicate the mechanism responsible for the declining rate. Fitting corrosion damage to a power law equation of the form

(2) $$y=at^{n}$$

where *y* is the measure of corrosion damage and *t* is the exposure time and the constants *a* and *n* are determined by the fitting process [7, 8, 20, 21]. In the case where corrosion damage is constant with respect to time, the exponent, *n*, will be one and in the case of a growing barrier film *n* will be 0.5 and other postulated rate limiting mechanisms may yield other values. An *n*-value greater than one would indicate that the corrosion rate increased with exposure time. While the nucleation of pitting after some incubation period longer than the first or second retrieval could result in *n*-values greater than 1, pit nucleation times are usually very much shorter than these exposure times and *n*-values between 0 and 1 are frequently observed for corrosion damage rates [21].

This time dependence was recognized in the original NBS studies and they examine this trend by linear regression of the equation

**Fig. 13:**
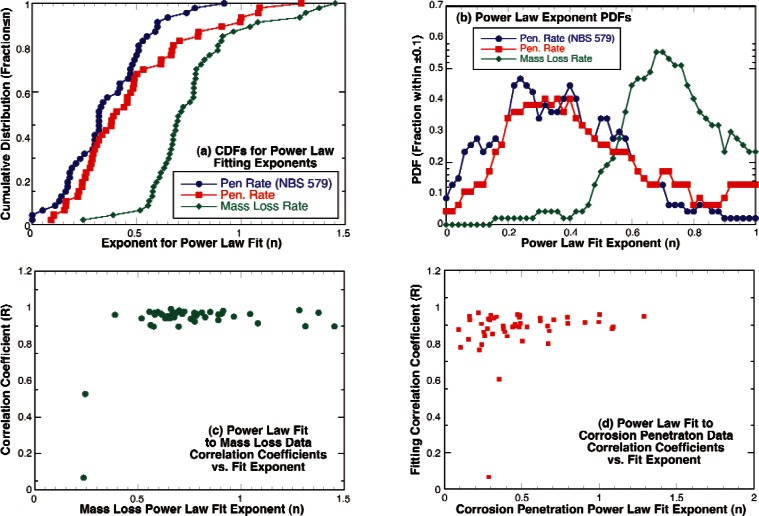
Graphic presentation of the results of fitting mass loss and corrosion penetration measurements for each site to a power law equation: (a) cumulative distribution functions for exponents determine by fit, (b) probability distribution functions for exponents determined by fit, (c) variation of correlation coefficients for mass loss with exponent of fit, and (d) variation of the correlation coefficients for penetration with exponent of fit.

(3) log(y)=log(a)+nlog(t)

with *y* equal to the two sample average maximum penetration for the exposure time (*t*). For this report, the measurements from each site for mass loss and corrosion penetration were fit to Eq. (2) using commercial software for iterative non-linear curve fitting that uses a Levenberg-Marquardt algorithm for estimating successive iterations until the squares of the errors reach a minimum [22]. The use of a non-linear curve fitting routine allows for inclusion of the initial (zero exposure time, zero damage) data points in the curve fits that cannot be included in a linear regression of Eq. (3). The results of these fits are shown in Fig. 13 along with the results reported by in the NBS underground corrosion reports [7, 8] for linear regression per Eq. (3). Figure 13(a) is a CDF for the fitting exponent (*n*) and Figure 13(b) is a PDF for this same parameter. By examining these figures, it can be seen that the corrosion penetration rate and the mass loss rate exhibit significantly different time dependences. That is, the corrosion penetration rate tends to slow to a much greater extent with exposure time than the mass loss rate. This indicates that the corrosion penetration rate is being limited by the mass transport of cathodic reactants or anodic products through the corrosion products building up at the pit while the rate limiting processes governing the mass loss rate and not facing the same restrictions. This also explains the trends shown in Figs. 9(d)–9(f). Figures 13(c) and 13(d) show the correlation coefficients determined for the curve fits for the mass loss data and the corrosion penetrations respectively and these figures show that with the exception of two points, most of the correlation coefficients for mass loss were above 0.9 and above 0.8 for the penetration data. These figures also show that there is no clear trend in the correlation coefficients with the value determined for the power law exponent (*n*).

## 6. Conclusions

After extensive examination and reexaminations of the data presented in the NBS studies of underground corrosion it is concluded that while equations for the estimation of corrosion damage distributions and rates can be developed from these data, that the scatter inherent in these models is considerable larger than it could be and that this will always limit the ability of predictions to be made from models based on this data. The scatter in these measurements is the result of the state-of-the-art at the time the study was conducted and the limitations of budget and size of the project. The data indicate that more complete datasets with soil property measurements reflecting the properties of the soil and ground water directly in contact with the samples including annual and seasonal variations and obtained with statistical analysis of the results considered during the design of the experimental program would greatly reduce this scatter and enable more representative predictions.
